# Integrating a Mobile App to Enhance Atrial Fibrillation Care: Key Insights From an Implementation Study Guided by the Consolidated Framework for Implementation Research

**DOI:** 10.2196/66815

**Published:** 2025-04-30

**Authors:** Sumudu Hewage, Sanjeewa Kularatna, William Parsonage, Tomos Walters, Steven McPhail, David Brain, Michelle J Allen

**Affiliations:** 1 Australian Centre for Health Services Innovation Queensland University of Technology Kelvin Grove Australia; 2 Health Services and Systems Research Duke-NUS Medical School Singapore Singapore; 3 National Heart Research Institute Singapore National Heart Centre Singapore Singapore; 4 Cardiology Department Royal Brisbane and Women's Hospital Brisbane Australia; 5 Queensland Cardiovascular Group Brisbane Australia; 6 Faculty of Medicine University of Queensland Brisbane Australia; 7 Digital Health and Informatics Directorate Metro South Health Brisbane Australia

**Keywords:** mobile health apps, digital health integration, health care innovation, technology adoption, cardiac rehabilitation, lifestyle modification, implementation science, consolidated framework for implementation framework, mHealth, mobile health, app, digital health, smartphone, eHealth, telehealth, telemedicine, digital, technology, CFIR, implementation research, cardiac, rehabilitation, cardiology, atrial fibrillation, Australia, interview

## Abstract

**Background:**

Despite the growing use of mobile health apps in managing chronic heart disease, their integration into routine care remains challenging due to dynamic, context-specific barriers.

**Objective:**

This study aimed to identify the key enablers and challenges of implementing a mobile app for cardiac rehabilitation and healthy lifestyles in patients with atrial fibrillation at an Australian cardiology clinic.

**Methods:**

We interviewed both clinicians and patients to understand their perspectives about the mobile app and what factors affected the implementation. The two semistructured interview guides used, one for clinicians and one for patients, were developed based on the Consolidated Framework for Implementation Research (CFIR) and nonadoption abandonment, scale-up, spread, and sustainability complexity assessment tool. All interviews were recorded and transcribed, and the transcripts were analyzed inductively to generate codes using a constructionist perspective. These codes were subsequently mapped onto the constructs within the CFIR across its five domains. This framework analysis was followed by examining the interconnections among the constructs to understand their collective impact on the implementation process, thereby identifying key enablers and challenges for the integration efforts.

**Results:**

We interviewed 24 participants including 18 patients, whose mean age was 69 (SD 9.2) years, and 6 clinicians, comprising 4 specialist cardiac electrophysiologists and 2 nurses. Patient engagement with the app varied: 3 participants completed the cardiac rehabilitation plan, 1 participant was still actively engaged, 2 participants had partial use, 10 participants downloaded but never used the app, and 2 participants did not download the app. We identified a complex interplay between key determinants across all five CFIR domains, collectively impacting two main elements in the implementation process: (1) acceptability and user engagement with the app and (2) the clinic’s implementation readiness. The app was more likely to be accepted and used by patients who needed to establish healthy lifestyle habits. Those with established healthy lifestyle habits did not indicate that the app provided sufficient added value to justify adoption. Interoperability with other devices and design issues, for example, limited customization options, also negatively impacted the uptake. The clinic’s implementation readiness was limited by various challenges including limited staff availability, insufficient internal communication processes, the absence of an implementation evaluation plan, and lack of clarity around who is funding the app’s use beyond the initial trial. Despite the clinician’s overall inclination toward technology use, diverse opinions on the evidence for short-term cardiac rehabilitation programs in atrial fibrillation critically reduced their commitment to app integration.

**Conclusions:**

Mobile health apps have seen rapid expansion and offer clear benefits, yet their integration into complex health systems remains challenging. Whilst our findings are from a single app implementation, they highlight the importance of embedding contextual analysis and proactive strategic planning in the integration process.

## Introduction

Atrial fibrillation, a chronic and multimorbid heart condition, often necessitates lifelong management. Recent figures indicate its rapid rise to epidemic proportions globally [1]. In Australia, atrial fibrillation–related hospitalizations surged by 295% between 1993 and 2013, far exceeding the 73% rise for myocardial infarction and 39% for heart failure during the same period. The financial impact was equally striking, with atrial fibrillation costs escalating by 479%, more than double that of myocardial infarction or heart failure [2,3].

International clinical practice guidelines endorse the implementation of digital health technologies, including mobile apps, to enhance the provision of integrated care for patients with atrial fibrillation [4-6]. Mobile app use has been shown to be effective in ongoing therapeutic monitoring and management [7-9], medication adherence [10-12], and in delivering health information to patients and carers [10-12] in atrial fibrillation.

Health-related mobile apps available to consumers in major app stores now exceed 350,000, with over 90,000 new digital health apps added in 2020 alone. This equates to the addition of more than 250 apps per day. Nearly half of all disease-specific apps are focused on mental health, diabetes, and cardiovascular disease [13]. With over 311 million active users, these apps generated revenue of US $3.4 billion for the industry in 2023, marking a 10% increase compared to the previous year [14].

Despite this accelerated uptake of mobile health apps, their successful integration into routine patient care has been challenging [15-17]. Health systems are inherently complex, and the reliability of technology can vary across contexts [18]. Such complex health systems must adapt in various ways to accommodate even seemingly simple technologies, which could make the implementation process formidable. Growing evidence also indicates that different user characteristics play a crucial role in determining the success of digital health interventions [19-21].

While the potential benefits of mobile health apps in cardiac rehabilitation are widely recognized [22,23], there is limited understanding of the context-specific barriers and facilitators that influence their integration within clinical settings. In particular, factors such as clinician engagement, implementation readiness, and the role of user characteristics remain underexplored, representing a critical gap in the evidence base for effective implementation strategies. Considering the global shift toward digital health and the endorsement of app integration in clinical guidelines to enhance patient care, this gap hinders the realization of mobile health apps’ full potential in chronic disease management. Implementation science provides a robust methodological approach to address this gap, as it accounts for the dynamic factors that shape real-world implementation outcomes [24,25]. Theories, models, and frameworks in this field offer structured methods for systematically examining the determinants of successful adoption and integration [26].

In this study, we applied two implementation science frameworks, the updated Consolidated Framework for Implementation Research (CFIR), one of the most cited frameworks in implementation research [27], and the nonadoption abandonment, scale-up, spread, and sustainability—complexity assessment tool, a deterministic framework with a focus on digital health technology [18]. Using these frameworks, this study aimed to identify key enablers and challenges in integrating a mobile health app designed for cardiac rehabilitation and healthy lifestyle promotion among patients with atrial fibrillation at a private cardiology clinic in Australia. In doing so, we addressed the limited understanding of context-specific barriers and facilitators that remain underexplored in digital health integration efforts in health care.

## Methods

### Study Design

This study is an implementation evaluation using qualitative interviews to explore the factors determining implementation success, including the enablers and challenges to the app’s uptake and integration into routine care from both patient and clinician perspectives.

### Study Context

The clinic in this study is a private, independent facility specializing in heart rhythm disorders, located within two major private hospitals in metropolitan Queensland. In the Australian context, private clinics are independently funded through patient payments and private health insurance, offering flexibility and quicker access to specialized care outside of the public health care system. It serves patients from local urban populations, as well as those referred from regional and remote areas across the state, spanning over 1.73 million square kilometers [28]. Telehealth facilities are regularly used for consultations irrespective of patients’ residence, and the clinic currently uses both its website and social media to disseminate health education and promote services. A web-based software tool is already available for clinicians to collect and analyze patient data for monitoring clinical progress.

### Innovation

Introduced in April 2023, the mobile app examined in this study is primarily designed to enhance patient engagement in a 12-week cardiac rehabilitation program following catheter ablation for atrial fibrillation. The app allows users to monitor vital signs, record symptoms, and upload electrocardiogram images but does not provide feedback on entered data. It features medication reminders, video and reading educational materials, and guided relaxation videos. Available exclusively to clinic patients free of charge, the app’s effectiveness in this atrial fibrillation cohort had not been formally evaluated. Identifying and managing risk factors are Class I (evidence or general agreement that a treatment or procedure is beneficial and effective) and Level B (data derived from a single randomized clinical trial or large nonrandomized studies) recommendations in atrial fibrillation clinical practice guidelines [29,30].

**Figure 1:**
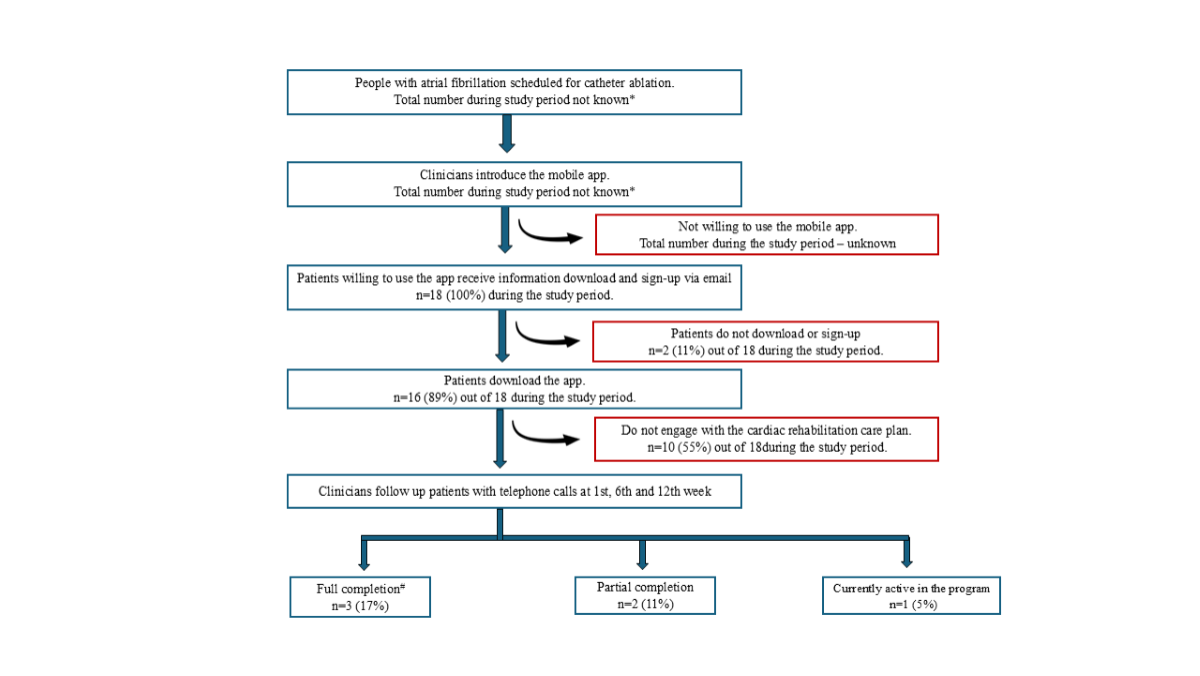
Flowchart depicting possible patient progression from app introduction to care plan completion. *Not a part of routine data collection; #Full completion of the cardiac rehabilitation care plan is defined as either completing the 12-week scheduled plan or satisfying the specialist’s criteria for completion.

Patients scheduled for catheter ablation are introduced to the app by clinicians near the procedure date. Interested patients receive email instructions for downloading and signing up. Nurses then use the clinician portal to view enrolled patients and set up a cardiac rehabilitation care plan with clinical data. Post the procedure, nurses promote app use during follow-up calls at 1, 6, and 12 weeks. The care plan is completed at 12 weeks or when the specialist cardiac electrophysiologist is satisfied, but patients can request early discharge. Clinicians access patient data through their portal, and specialists are anticipated to review patients’ progress before the 12-week consultation. Figure 1 illustrates this process flow.

### Participants and Data Collection

Participants included eligible patients who had been offered the app after catheter ablation, regardless of whether they used it, and clinical staff at the clinic involved in implementing the app. Patients with cognitive and language-related disabilities that might hinder their ability to express opinions were excluded. Clinic nurses informed eligible patients about the study during planned follow-up care calls (6 weeks after ablation) and sent them a participant information sheet. Interested patients were contacted by the research team via email or text to schedule interviews. Nonresponders were identified after no reply to an initial invitation and a reminder after 3 days. Telephone interviews were conducted with verbal consent, and patient participants received AUD 20 (~US $12) gift voucher upon completion.

All specialist cardiac electrophysiologists and nurses involved were eligible to participate, with no specific exclusion criteria. They were initially contacted via email to introduce the study, followed by scheduling interviews with the research team. Clinician interviews were conducted face-to-face, and written consent was obtained.

SH conducted all interviews using semistructured questionnaires (Multimedia Appendix 1), which were developed based on the CFIR [31] and nonadoption abandonment, scale-up, spread, and sustainability—complexity assessment tool interview guide [32]. All interviews took place between April 30 and July 1, 2024.

### Data Analysis

**Figure 2:**
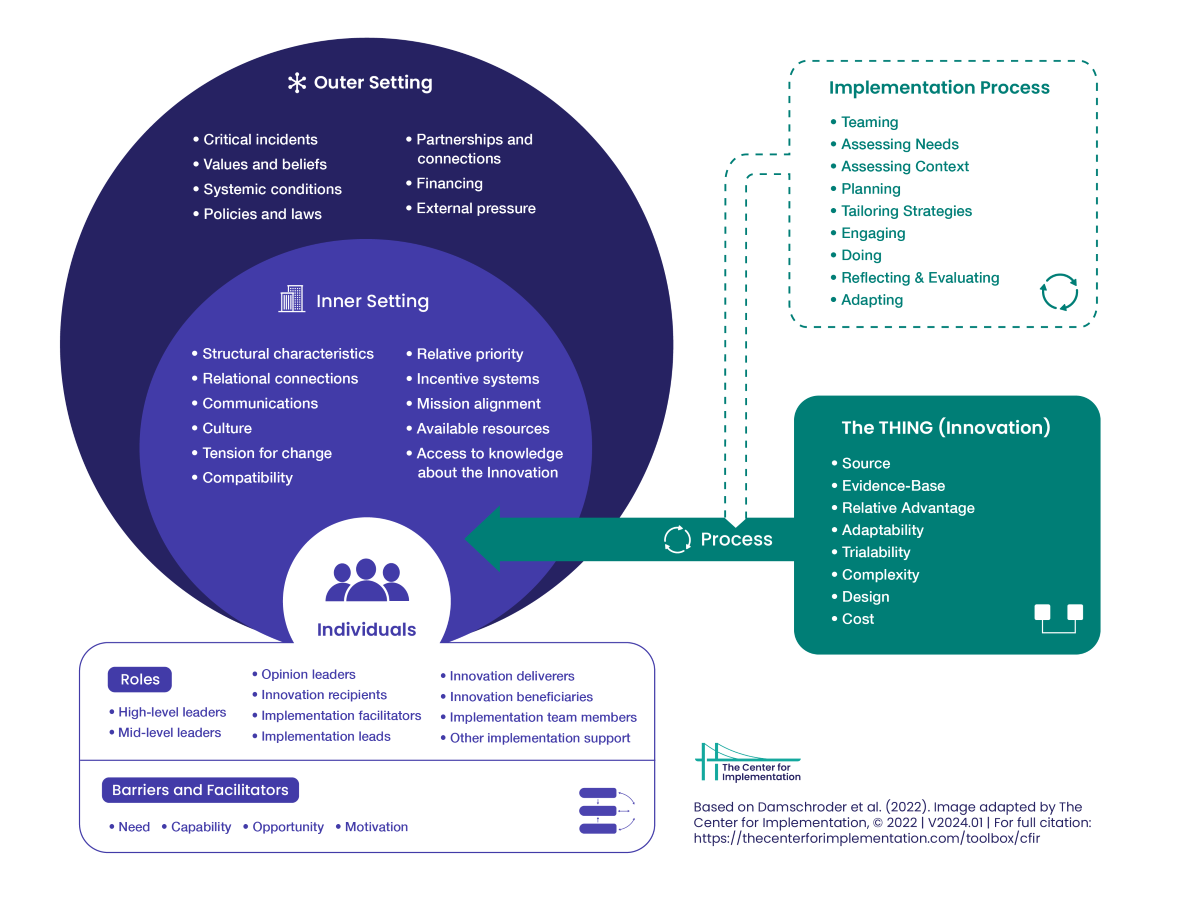
The Consolidated Framework for Implementation Research (CFIR) with its five domains. Adapted from Damschroder, LJ et al [34].

Recorded interviews were transcribed verbatim using the transcription function in Word (Microsoft). The transcripts were then thoroughly reviewed to confirm accuracy and gain familiarity with the content. Meaningful segments of text were identified, condensed, abstracted, and coded. A constructionist view was adopted during this process, allowing for the exploration of diverse interpretations of stakeholders’ experiences with the mobile app, considering personal, organizational, and contextual factors influencing perceptions of enablers and barriers [33]. The identified codes were then mapped onto the 31 constructs within the five domains of the CFIR: Innovation, Outer Setting, Inner Setting, Individuals, and Implementation Process (Figure 2 [34]).

Each code was initially assigned to a single construct within a domain. In cases where both analysts concurred that a code could fit into multiple constructs across domains, the analysts discussed and reached a consensus on the most appropriate construct. Following this process, codes were read and reread to understand their potential influence on the construct, as well as on the implementation process. Based on this impact, they were categorized as enablers or barriers to mobile app integration into routine health care services. SH and MJA undertook the iterative analytic process collaboratively.

The results of this analysis are presented as a text for each CFIR domain, supported by quotes extracted from interview transcripts. Each quote is assigned a unique ID to ensure participant confidentiality. These IDs distinguish between patients and clinicians but do not specify whether the clinician is a doctor or a nurse to prevent potential identification due to small numbers. Additionally, the IDs do not reveal the management roles of doctors within the organization to further preserve anonymity.

### Positionality of the Research Team

SH has over 8 years of experience as a medical doctor and public health researcher. MJA is an implementation scientist and health services researcher, with a public health background in government and not-for-profit sectors. The remaining members of the research team—WP, TW, SM, SK, and DB—include specialist cardiac electrophysiologists, health services researchers, and health economists, who also contributed to the interpretation of the findings. While SH and SM bring both insider and outsider perspectives due to their experience in cardiology clinical settings and rehabilitation programs, MJA, SK, and DB provided interpretations from an outsider’s perspective, having no direct experience in cardiology. In contrast, WP and TW, with experience in this clinic, contributed insider perspectives.

### Ethical Considerations

The study was conducted in conformance with the principles of the National Statement on Ethical Conduct in Human Research [35]. Ethics and administrative approvals were obtained from the human research ethics committee of the Queensland University of Technology and the clinic administration prior to commencing data collection.

## Results

### Overview

Of the 26 eligible patient participants invited to join the study, 18 participants accepted and were interviewed, yielding a response rate of 69%. The mean age of the participants was 69 (SD 9.20) years and 10 (55%) were male participants. Patients exhibited varied use patterns, ranging from completing the cardiac rehabilitation care plan and regularly entering data to never downloading the app (Table 1).

All 6 clinicians involved in the mobile app implementation were interviewed, comprising 2 nurses and 4 specialist cardiac electrophysiologists. The 4 specialists, in addition to their clinical duties, also serve in high and midlevel management positions within the clinic. This dual role allowed them to offer insights shaped by both clinical practice and managerial oversight.

**Table 1:** Mobile app use pattern by demographic and illness-related data (n=18).

			App use pattern								
			Cardiac rehabilitation care plan fully completed^a^ (n=3), n (%)^b^		Currently actively using one or more app features (n=1), n (%)^b^		Used at least one app feature more than once and stopped (n=2), n (%)^b^		Downloaded the app but never used any app features (n=10), n (%)^b^		Received the link but did not download the app (n=2), n (%)^b^
**Age (years)**											
	<50 (n=1)	—^c^		—		1 (6)		—		—	
	50-59 (n=2)	—		—		—		1 (6)		1 (6)	
	60-69 (n=6)	2 (11)		—		1 (6)		3 (17)		—	
	70-79 (n=6)	1 (6)		—		—		4 (22)		1 (6)	
	>80 (n=3)	—		1 (6)		—		2 (11)		—	
**Sex**											
	Male (n=10)	2 (11)		—		—		6 (33)		2 (11)	
	Female (n=8)	1 (6)		1 (6)		2 (11)		4 (22)		—	
**Duration of atrial fibrillation (years)**											
	<1 (n=4)	1 (6)		—		—		3 (17)		—	
	1-4 (n=5)	1 (6)		—		1 (6)		2 (11)		1 (6)	
	5-9 (n=6)	—		1 (6)		1 (6)		3 (17)		1 (6)	
	>10 (n=3)	1 (6)		—		—		2 (11)		—	

^a^Full completion of the cardiac rehabilitation care plan is defined by the clinic as either completing the 12-week plan or satisfying the specialist’s criteria for completion.

^b^Percentage values represent the proportion of the total number of participants (N = 18).

^c^Not applicable.

### Framework Analysis

#### Overview

The results are presented as a narrative for each CFIR domain: Intervention Characteristics, Outer Setting, Inner Setting, Characteristics of Individuals, and Process of Implementation. The full analyses including CFIR domains matched with corresponding codes and quotes are detailed in Multimedia Appendix 2.

#### Innovation

Of the constructs in the Innovation domain, our codes encompassed complexity, evidence source, relative advantage, design, and cost. The app’s functionality relies on several external factors, including the availability of a smartphone and internet connectivity, but clinicians reported no refusals due to these issues. Patients found the app easy to download, sign up for, and navigate.

Although there was agreement on the benefits of lifestyle modification for patients with cardiac diseases more broadly, differing opinions about the effectiveness of a 12-week cardiac rehabilitation plan specifically for atrial fibrillation among clinicians were apparent.

Before we even got the app and when we started interacting with [app developers] to build it [the app], I wondered how it would fit, to be honest. Because AF [atrial fibrillation] in comparison to a rehab program where we rehab a person who’s had the heart attack and stent, AF is this lingering chronic condition that comes and goes, and people don’t stay motivated in apps for very long relative to the length of time they’re going to have the condition. If so, I wonder how a 12-week program post an AF ablation will transpire into a long-term outcome, so we’ll wait and see. But I doubt we’ll have many patients motivated to continue.

Clinician 04

From patients’ perspectives, those accustomed to smartphones’ built-in functionalities and wearables like smartwatches found these existing tools more advantageous due to their ease of use and established integration into their routines, consequently resulting in reduced app usage.

I also wear a smartwatch and I use that to monitor things like that [steps and pulse rate]. So rather than using the app on my phone, it was just quicker and easy just to have a quick look at my watch and you know, do a little download, all that sort of thing.

Patient 08

In contrast, patients who did not use smart devices or lacked alternative strategies found the app to be highly beneficial.

I made my own [medical records], and it was very clumsy. So, when I realized it was all in one app where I can record my medication as well, and it’s got all sorts of things. I do my weight, my blood pressure, pulse rate, and my medication, all record(ed) in there and it’s easy to have a look back and compare on how it was going, you know, so it’s good.

Patient 07

Additionally, poor app interoperability hindered data integration from certain types of smartphones and was highlighted as a barrier to engagement. Other frequently noted design issues included limited options in drop-down menus, the need to delete and re-enter data when ablation was postponed due to the inability to modify the ablation date, and inconsistent date formatting.

In the innovation cost, we investigated patients’ attitudes toward paying out of pocket for the app. There was marked hesitation to incur such expenses, with participants also advocating for mobile apps designed for routine health care to be freely accessible to patients.

No [I will not pay for this app]. It should be [a] free app. It’s about postoperative care [therefore] it should be free. That should be part of the rehab process. And I know how cheap apps are to manage. You roll it [app] out, it gets it there. It’s actually really cost-effective unless the point is to do business.

Patient 04

We also examined users’ concerns about the app’s data security. None of the patients viewed the app as a potential threat to their data privacy. This lack of concern may have been underpinned by a limited understanding of potential data threats and high trust in the app providers.

I know we are living in a very scummy world, but yeah, I mean if someone wants to know about my heart rate and how many beeps it’s doing a minute, I don’t care. When it comes to bank account details and credit card details, that’s a different story.

Patient 14

I’m not concerned about data privacy because you have to register, so it’s not like a public app. I had to register with the clinic.

Patient 02

#### Outer Setting

We defined our outer setting as the general public and relevant stakeholders beyond the clinic setting, including the government, health technology companies, and broader hospital and health systems. We analyzed the constructs of local conditions, market pressure, and financing that were prominent in this domain.

All clinician participants concurred that the broader system holds a positive attitude toward the use of digital technology in health care. The public’s perception was similarly seen as favorable, particularly in terms of accepting technology integration into routine health care, a sentiment that has grown stronger following the COVID-19 pandemic.

I think it [mobile health technology] is very valuable. I actually think it’s a game changer conceptually in how we’re managing patients and if that can then become more broadly used, I think you do start to have impact on services, particularly if you can make patients less reliant to actually coming into a health center.

Clinician 03

Clinicians also observed that commercial entities in the digital health care market are rapidly influencing the cardiology landscape and expressed optimism that this trend will likely continue to grow.

This [digital technology] is the health market across. And this is growing, it’s getting bigger and bigger every year. [participant shows a web page for a professional conference on the computer monitor] this is huge … They’ve now got a whole separate conference which is a digital health cardiac conference. [participant points at logos on the screen] These are sponsors and exhibitors and you’ll recognize a lot of them. And they’ve got a journal specifically looking at digital health.

Clinician 04

There’s already support for a number of health districts around Australia. [hospital and health services] are already providing app-based rehab.

Clinician 06

However, there was a perception that the government is not readily facilitating the integration of health technology through funding support, presenting a key challenge. Clinicians have expressed concerns about delays in necessary reforms and reimbursements.

Governments? Very slow. Very slow to reform … I mean, just to get remote monitoring as a refunded item number for all of our pacemakers took years and years and years of negotiation … They [government] do ultimately respond to, let’s say public pressure. And the more something is normalized as the way for it to be done and utilized, the more they’re going to have to fund it.

Clinician 05

#### Inner Setting

The cardiology clinic, considered the “inner setting” for this study, consists of 4 cardiac electrophysiologists specializing in cardiac rhythm management and ablation procedures; and two nurse practitioners who handle patient enrollment, monitoring, medication concerns, and care plan management. Our analysis was based on the constructs of culture (recipient-centeredness and learning-centeredness), structural characteristics (information technology infrastructure and work infrastructure), compatibility with workflows, relational connections, communication, tension for change, and resource availability.

Work culture within the clinic was strongly positive, embracing a holistic approach to patient care. The clinicians also showed an interest in data-based learning, indicating the learning-centeredness of the setting.

I really am interested in is the analysis of the data and trying to show that these sorts of strategies [mobile apps] work, that there is benefit in it for a population and then you know, publish and expand that role.

Clinician 05

The clinic had a strong information technology infrastructure with regular use of telehealth facilities, web-based software tools, and social media platforms to promote its services. This inclination toward adopting cutting-edge technologies is evident across all aspects of care, from delivering health education to using the latest medical innovations, such as ablation devices.

As an institute, we want access to the latest technologies, be it for ablations devices, whatever we have.

Clinician 05

This was identified as a key enabler for the app’s implementation, as clinicians were inherently supportive of using technology to deliver and improve health services. Despite this interest, the impact of the app on routine clinic operations remains uncertain. Notably, only one clinician reported integrating patient-entered app data into their regular clinical management.

Probably in about 50% of those who’ve been enrolled, I would have used it, at least to some extent. Particularly sort of when I’m about to see them.

Clinician 03

The proactive reorganization of clinicians’ routine work enabled more time to engage with implementation activities, indicating a positive work infrastructure.

So, they [clinic administration] have altered some of our other things that we have to do. And that probably will help free us up right a bit more.

Clinician 01

However, the implementation process occasionally fell short in specific areas irrespective of these efforts. For instance, the absence of a system to flag every patient registering for ablation procedures sometimes resulted in patients being missed and not introduced to the app before the procedure.

There is a big workflow issue we have at the moment. If we’re not notified that a patient’s going for ablation, we don’t know to call or send out the education materials. So, unless we’re monitoring all the doctor’s diaries and all the different sites, sometimes we can just miss them entirely.

Clinician 02

While the relational connections, both formal and informal, within the clinic, were of high quality, the absence of an administrative hierarchy in the private clinic was recognized as a challenge to driving the implementation process.

One of the issues I guess in sort of private practice context compared to a public departmental context is we don’t have a hierarchy. So, we don’t have a structure in that sense, you know there’s no director who sets expectations and creates a framework that is applied to all patients. So, you know, what I do might be a bit different to what Dr [name] does, or what Dr [name] does.

Clinician 03

We noted varying levels of awareness among the implementation team regarding the app. Overall poor communication, both general and specific to the mobile app, posed challenges to implementation.

Well, that [the app] may have been developed as part of the clinic, but I hadn’t been involved too much in the development of that. That’s [the app is now available for patients to use] actually news to me. I wasn’t actually aware that we had a specific AF thing ready for market, so to speak.

Clinician 06

Doubts about the efficacy, a lack of institutional hierarchy mandating the implementation, and its recent introduction resulted in a low tension to change, leading to a lower priority for the app within the time-constrained consultations.

I must admit I don’t do a lot of talking to them [patients] about the app because the app is something that we only really started to push probably this year.

Clinician 04

Crucial challenges were identified within the resources construct, primarily due to manpower and ongoing funding issues. The current initial phase of the app’s implementation is supported by research funding from external sources, but long-term financial sustainability remains unplanned. Stakeholders’ concerns about profitability further complicate internal funding considerations.

I think one of the other biggest hurdles that I foresee is how to monetize these sorts of things, and it’s all expensive. At the moment we’re paying for it from research funding. So, we’ve funded the first 100 patients for the App. But we won’t be able to keep funding beyond that. The financial resources aren’t there.

Clinician 05

#### Individuals

The two groups we considered within the domain “Individuals” are the innovation users—patients with atrial fibrillation who have been introduced to the mobile app following the ablation procedure, and innovation deliverers—clinicians within the clinic. Constructs of need, motivation, capability, and opportunity in the Individuals Characteristics subdomain were prominent.

The need for a lifestyle modification app among patients was largely driven by their current habits or strategies in this area. Not unexpectedly, participants without prior established healthy habits found the app more useful, while those already practicing a healthy lifestyle viewed it as redundant.

With the exercise, I already have a strong routine of going for a walk every day. I had a regular pattern of taking the medication, so I didn’t need reminders for that. I’ve had other apps I use for mindfulness or meditation. I go to yoga, and I don’t remember finding it [app] helpful in that way. There’s lots of different ways to develop habits that are healthy, and I had already done that.

Patient 05

Despite recognizing the app’s potential to meet specific needs, a lack of engagement persisted in some, reportedly attributable to their lack of motivation.

I think the problem was me. I’m just lazy. I think it [app] will be very helpful to organized people, you know. I’m one of those people that don’t have a routine. I never have, really.

Patient 13

I’m interested in being healthy. Don’t get me wrong, but I really can’t be bothered to watch videos from doctors telling me not to drink alcohol or not to have too much sugar or anything like that.

Patient 03

Clinicians’ observations confirmed that maintaining patients’ motivation throughout the duration of the cardiac rehabilitation care plan was challenging*.*

I’d say the majority [of patients] don’t [engage with the app] by 6 weeks. I mean, some are really good, really religious with it. They’re the ones that will generally carry it through. But a lot of the times they’ll sign up. Maybe they’ll do a week or two and then fall off, but most times they’ll sign up and not really put anything in.

Clinician 02

The capability of older patients to engage with the app, despite stereotypes suggesting digital aversion, was found to be high. On the other hand, younger patients were reported to lack the opportunity to engage with the app even though they possess the required knowledge and skills.

You would think that the older they are, the less likely they are [to use digital technologies], but that’s not necessarily the case. They [those who engage well with the app] are definitely sort of the older cohort, sort of 60 to 70 population, I’d say they’re more signing up to it. We do actually have quite a few young people that come through and have ablations. Almost always, they won’t sign up, and I don’t know whether that’s to do with lifestyle like they’re back into work or they’ve got young kids, they just don’t have the time.

Clinician 02

From the clinicians’ perspective, opportunities to introduce the app to patients were commonly missed by specialists. These stemmed from time constraints, inconsistent prioritization of the app, and procedural barriers, such as patients registering for ablation after a waiting period and having only brief interactions with specialists before the procedure. These had significant implications because specialist endorsement appeared to facilitate the adoption by patients.

If the doctors are peddling it [app introduction], they [patients] are much more open to the idea.

Clinician 01

*I probably should have it as a sort of mental tick box to say. One of the things I should specifically and explicitly mentioned to patients is you’ll be offered the use of this app by our nurses. Yeah, and sort of talk about in these particular virtues and what we’re hoping or how it, how we’re hoping it’llhelp them*. [Clinician 03]

#### Implementation Process

In this domain, we evaluated the implementation team’s efforts to facilitate the app’s introduction, adoption, and sustained use by patients. Our codes aligned with the constructs planning, reflecting and evaluating the implementation, tailoring strategies, engaging innovation recipients, and assessing the needs of the recipients in analyzing the domain of the implementation process.

An enabling factor was the identification of clear roles and responsibilities within the implementation team. However, processes for reflecting and evaluating were not outlined or defined in advance, leading to lost opportunities for improvement.

I think in the context of our clinic, the things that would be helpful would be to develop consistency between the various clinicians in terms of how patients are introduced to it ... perhaps a more structured framework to support the nurses who are practically managing on a day-to-day basis and a practical framework for collecting the data and using the data. So, you know we have it, we’re using it. We’re perhaps not using it as efficiently or effectively as we could or should.

Clinician 03

The initial implementation plan was prepared with the involvement of the team, but it was necessary to tailor key strategies due to human resource constraints. For example, clinicians had to reduce the frequency of follow-up calls from weekly to 1, 6, and 12 weeks post procedure due to staff unavailability. Both patients and clinicians indicated that follow-up frequency impacted app adoption.

We [clinicians] knew that that [weekly follow-up calls] wasn’t going to work for us because we don’t have the manpower. With [ name], and [name], I think at that [designing] stage, we discussed as to how we could fit that to ... to around us.

Clinician] 01

I agree with that other patients’ criticism where there would be a lot more value to it if we are checking more frequently every week.

Clinician 02

Blanket prescribing of the app without considering individual patient needs for lifestyle modification, and a lack of (detail or thorough explanation) about app features, appears to have negatively impacted adoption and sustained use.

...it sounds to me as though they didn’t sell the app well enough to me. Had they explained some of the features and benefits a little more, I might have used it more ... the first thing I started to do was put in all my medicines and then got all these bloody messages, you know which annoyed me, so I thought [not to use the app]. Well, I’ve still got the app in my phone. I might have another look at it.

Patient 14

### Collective Analysis

**Figure 3:**
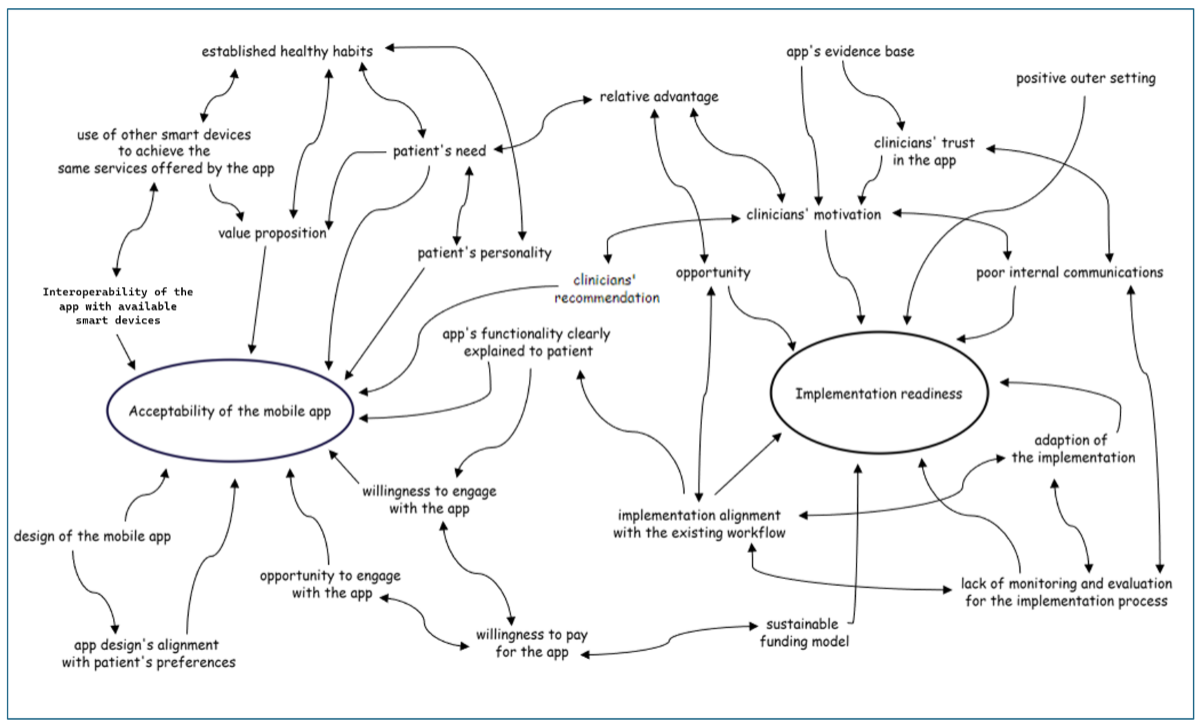
Loop diagram illustrating the interconnections between factors across five CFIR domains and their collective impact on the app’s acceptability and implementation readiness of the clinic. CFIR: Consolidated Framework for Implementation Research.

A collective analysis of the codes revealed a complex interaction between key determinants across all five CFIR domains, impacting two main elements in the implementation process: (1) acceptability and sustained engagement, and (2) the clinic’s implementation readiness (Figure 3).

## Discussion

### Principal Findings

This study contributes to closing the knowledge gap in understanding the context-specific barriers and facilitators that influence the integration of digital health interventions in clinical practice, particularly in the implementation of mobile health apps for cardiac rehabilitation and lifestyle management. In summary, among the many app-related features, a simplified downloading process, intuitive design, and low complexity were key enablers of its acceptability and use, but manual data entry flaws and interoperability issues due to mobile phone type reduced user engagement. Several key patient characteristics also influenced app uptake. Those with already established healthy lifestyle habits (eg, regular exercise) were less likely to find the app useful, while personality traits such as motivation were also found to play a significant role in the adoption and sustained use of the app. Interestingly, older patients were more likely to engage with the app compared to younger patients. Clinicians expressed varied levels of trust in the app’s effectiveness, and patients’ unwillingness to pay for it posed a potential barrier to the program’s sustainability. A favorable digital health perception within the organization and shared values helped drive adoption, but challenges such as insufficient internal communication, resource constraints, and workflow misalignments hindered the successful integration of the app into routine care. Furthermore, missed opportunities by specialists to introduce the app, coupled with a lack of structured reflection and evaluation processes, contributed to lower adoption and sustained use rates. These findings highlight the necessity for unified commitment at both individual and institutional levels to fully harness the potential of digital health technologies.

This study revealed that factors across all CFIR domains collectively influenced both the app’s acceptability and the clinic’s implementation readiness. In digital health, acceptability is a fundamental yet complex concept, broadly defined as users’ attitudes toward, willingness to engage with, actual use of, or satisfaction with an innovation [36].

The adoption of innovation extends beyond simply introducing new technology; it also requires shifts in individual and group attitudes and behaviors [37]. Behavioral theories suggest that behavior change is a multifaceted process shaped by both individual readiness and external influences [38-40]. For instance, Rogers’ Diffusion of Innovations Theory explains that individuals adopt new technologies at different rates, with relative advantage, the perceived superiority of innovation over existing alternatives, being a key determinant of adoption [39]. Rogers further argues that while relative advantage can be measured in economic terms, factors such as social prestige, convenience, and satisfaction also play a critical role in the adoption process. According to his theory, the greater the perceived relative advantage, the faster an innovation is adopted [39]. Our findings align with this framework, as we identified several influential factors, both in this study and previous research [39,41-45] that could shape app adoption. These include pre-existing lifestyle habits, ease of integration into daily routines, personality traits (eg, being organized and motivated), and perceived value and satisfaction with the app. These results emphasize the importance of assessing patient needs and motivations prior to introducing digital health interventions including mobile apps. Prioritizing digitally ready, motivated users who are most likely to benefit from the intervention can enhance resource efficiency, as well as improve long-term engagement.

This also reinforces a critical insight for mobile app designers and implementers: achieving 100% adoption within the target population is unlikely, despite being the ideal goal. Furthermore, aligning with existing evidence [46], we found that digital literacy does not necessarily equate to digital readiness, as reflected in the lower adoption and engagement rates among younger participants. This challenges commonly accepted assumptions that younger, tech-savvy individuals are inherently more likely to engage with mobile health apps. Instead, our findings suggest that targeted implementation strategies, tailored to the specific characteristics and motivations of the intended users, may be more effective than relying on general demographic trends.

In addition, our results highlighted several app-related features that negatively influenced its acceptability, including limited interoperability and the need for manual data entry. These limiting factors are similarly seen to result in poor acceptability when health technology is perceived to be “more trouble than it is worth” [47,48], too time-consuming [49], unreliable [47], and does not fit the user’s workflow or routine [50,51]. Prior research has highlighted the potential benefit of the inclusion of features such as professional or peer interaction [41,52,53], passive monitoring over manual input [54], and advanced feedback mechanisms [40] to enhance app adoption and sustained use. To improve implementation, future iterations of the app should prioritize seamless integration with existing clinical systems, as well as reduce manual workload. Additionally, incorporating automated data capture and real-time feedback tailored to individual patient needs could further support engagement and long-term adherence. Considering these features during the app development stage or obtaining inputs from the specific target population on their preferences for these features could prove advantageous [55].

Readiness for implementation is a multifaceted construct in implementation science, encompassing stakeholders’ willingness and capacity to adopt new practices [56-58]. Commonly acknowledged individual factors influencing readiness include attitudes, commitment, and self-efficacy [56], and organizational-level factors influencing readiness include climate and resources [59]. At an individual level, the clinic’s readiness was limited by mixed attitudes toward the evidence for short-term app-based exercise programs in atrial fibrillation, which, in turn, influenced clinicians’ commitment to introducing the app during time-restricted consultations with patients. This also impacted uptake as patients were more inclined to adopt and engage with the app when their specialist endorsed it [60-62].

Furthermore, only one clinician in this study regularly used patient-entered app data for clinical management. Clinicians have been shown to adopt new technology when its benefits to their professional roles are evident [47,48]. Limited app introduction and use of collected data highlight a clear gap in clinician buy-in in this study. Gaining buy-in from clinicians, particularly around the evidence base and its value-add to clinical management, is a critical exercise in any health technology implementation [63]. This challenge of gaining clinician buy-in can be a complicated endeavor with an absence of consensus on what defines effectiveness in digital health [64]. At present, lack of scientific validation is a common problem among mobile health apps [65,66].

Engaging health care professionals and consumers early in the design process can help ensure that the intervention (both the app and its implementation) is seamlessly integrated into clinical workflows, its outputs are effectively used to enhance patient care, and it delivers meaningful value to patients. Additionally, establishing a clear evidence base through pilot testing, iterative feedback, and real-world feasibility studies should be prioritized from the outset to build confidence in the app’s effectiveness. These factors are critical for improving clinician buy-in and facilitating successful implementation.

At an organizational level, limited resource availability, poor internal communication, no internal champion or organizational mandate, the absence of ongoing monitoring and evaluation, and the lack of a long-term funding model limited the effective integration of the app. Available literature highlights that proactive and strategic planning during the pre and peri-implementation phases can help mitigate some of these challenges [51,67,68]. Establishing clear leadership roles within the implementation team, securing sustainable funding early in the process, and integrating structured monitoring frameworks to regularly track progress and address emerging challenges are crucial for the successful integration of digital health interventions, including mobile apps, into routine practice. Proactive planning and continuous stakeholder engagement can further enhance adoption and long-term sustainability.

Prior literature has highlighted significant inertia and resistance to new technology adoption within health care settings [67,69] and these factors contribute to slower adoption of new technologies in health relative to many other industries [47,68,69]. We observed the scenario in this study setting to be different. The clinic demonstrated a proactive approach to adopting digital technology, a tendency likely influenced by the positive attitudes of the staff, particularly the leadership [48]. However, despite this inclination toward digital technology in the current study setting, the implementation process remains challenging. This learning highlights the importance of strategic planning even in environments with a strong predisposition toward digital innovation.

Another key finding from this study is the absence of a clear funding model for the mobile app, which, as the literature suggests, poses significant challenges for its long-term sustainability [67,68]. Funding through patient fees, while straightforward, may exacerbate health inequities if patients are unable or unwilling to pay as indicated by our participants. External funding from bodies like health insurers is a viable alternative, but it necessitates a robust evidence base demonstrating the app’s clinical- and cost-effectiveness. To ensure long-term success and equity in digital health tool implementation, health care organizations must prioritize resolving these challenges during the implementation planning stage.

While this study was conducted in a single private clinic, we acknowledge that findings may differ in public hospitals, or nonmetropolitan settings, where resources and infrastructure may be more limited, internet connectivity less reliable, and clinical workload more demanding, potentially creating additional barriers to digital health adoption. However, our key findings related to the app itself, user engagement, and clinic-level implementation factors remain highly relevant across different health care contexts. Additionally, conducting this study in a private clinic may have influenced aspects such as clinician autonomy in adopting digital tools, patient demographics with potentially higher digital literacy, and financial flexibility which could differ in publicly funded or nonmetropolitan settings. Factors like financial constraints and scalability challenges in larger health systems may also shape how similar interventions are implemented more broadly. Future research should explore these contextual differences to better inform implementation strategies across diverse health care environments.

### Strengths and Limitations

We interviewed approximately one-quarter of patients, 18 out of 68 total patients who signed up for a cardiac rehabilitation care plan until June 2024 since project inception, with varying app use patterns. Unfortunately, despite considerable attempts by the research team to secure access to patient-level app use data to quantitatively explore use patterns or analyze associations between use and participant characteristics, this was not possible. Data on login frequency and feature usage were also unavailable, limiting our analysis to qualitative methods due to reasons beyond our control.

Using an implementation science framework enhanced the systematic examination of the qualitative data, by adding an additional lens through which to consider the code categorization and theme generation. This process pressed the authors not only to consider the data through their normal inductive process but also which parts of the implementation were affected, and in what ways.

The broader implications of our findings may be limited due to several factors. The small sample size restricts the extent to which our results can be applied to wider populations, and the low number of patients who fully completed the cardiac rehabilitation program limits our ability to draw definitive conclusions about long-term patient engagement. However, the challenge of poor patient engagement with cardiac rehabilitation programs [70-72], as well as in the adoption and sustained use of mobile health apps for disease navigation [73,74], aligns with results observed in broader research. Future research with larger, more diverse samples is needed to further explore these findings and assess their applicability across different health care settings.

Additionally, the study was conducted in a private cardiology clinic, which may limit the generalizability to dissimilar settings, particularly to public hospital settings or rural and low-resource environments. However, many of the challenges and facilitators identified such as the need for clinician buy-in, streamlined workflows, and patient engagement strategies are relevant across various health care contexts. In public hospitals, resource constraints and higher patient volumes may necessitate additional support mechanisms, while rural and low-resource settings may require adaptations to address differing levels of digital maturity and access to care. Future research may seek to examine how these findings can be adapted and scaled across diverse clinical environments. Nonetheless, this study highlights the importance of integrating contextual analysis and proactive strategic planning to enhance the adaptability of such interventions.

### Conclusions

The expansion of digital technology in routine patient care is expected to accelerate globally. However, it is essential to recognize that digital technology serves only as one means to achieve broader objectives. Its success in reaching these objectives depends on various dynamic and contextual factors within the health system and care teams, as well as the target population. Unless these multifaceted and intertwined factors are assessed and addressed before and during the implementation, health technology integrations are more likely to fail than to succeed [75,76]. Therefore, we advocate for a proactive approach to assessing contextual challenges and strategically planning for their mitigation, as discussed in this study.

## Appendix

Multimedia Appendix 1 Semistructured guides for interviews with study participants.

Multimedia Appendix 2 Framework analysis of the qualitative dataset.

## Abbreviations

CFIR: Consolidated Framework for Implementation Research

## Data Availability

All interview quotes and analytic codes relevant to the framework analysis are presented in the Multimedia Appendix 2.
