# Supplementary material for: Integrating a Mobile App to Enhance Atrial Fibrillation Care: Key Insights From an Implementation Study Guided by the Consolidated Framework for Implementation Research
Source: J Med Internet Res. 2025 Apr 30;27:e66815. doi: 10.2196/66815 (PMC12079067; doi:10.2196/66815)
Supplement: Multimedia Appendix 1 [file jmir_v27i1e66815_app1.pdf]

Supplementary file

Integrating a mobile app to enhance atrial fibrillation care: key insights from an implementation study guided by the Consolidated Framework for Implementation Research (CFIR)

Sumudu Avanthi Hewage [1] , Sanjeewa Kularatna [1,2,3], William Parsonage [1,5], Tomos Walters [6,7], Steven M. McPhail [1,4], David Brain [1], Michelle J Allen [1] ,

[1] Australian Centre for Health Services Innovation and Centre for Healthcare Transformation, School of Public Health and Social Work, Queensland University of Technology, Australia.
[2] Health services and systems research, Duke-NUS Medical School, Singapore.
[3] National Heart Research Institute Singapore, National Heart Centre, Singapore
[4] Digital Health and Informatics Directorate, Metro South Health, Queensland, Australia
[5] Cardiology department, Royal Brisbane and Women's Hospital, Queensland, Australia
[6] Queensland Cardiovascular group, Queensland, Australia
[7] Faculty of Medicine, University of Queensland, Queensland, Australia

PART A: Semi structured guide for interviews with specialist doctors

1. Could you tell me a bit about **your role** in this clinic and walk me through the **process** of how you introduce the app to patients?
    - ***<u>any eligibility criteria</u>*** *for patients?*

2. What are some **opportunities and challenges** you face during this process of introducing the app to patients?

3. How do patients typically respond to this offer? Has the uptake been good?
    - Frequent **questions** patients ask?
    - **Reasons** for **not** taking up?

4. How do you expect patients to use the app as part of their healthcare journey?
    - What app features? How frequently?
    - Is there any possibility that the app could lead to **issues or harm** in terms of patient management?

5. Are patients **actually using** the app in **the way you thought** they would? How's your experience been with that?

6. Have patients **shared any feedback,** positive or negative, about their experience with the app?
    - positive
    - negative
    - **If no → do you ask them about it when you make the follow-up call at 12-week mark?**

7. How does the app help you manage your patients better?
    - What data?
    - When do you them?
    - For what purpose?

8. What makes it **easier or harder** for you to use the data from the app in managing your patients?

9. We will now move on to discuss some organisation related factors. What's your opinion about the AF Institute's **capacity** and **readiness** to implement this app in the long run?

10. Do you think the institute has put in enough effort and resources to make sure this app work well?

11. From an investment standpoint, is there a risk of **financial loss** associated with the app?
12. Do you believe that the **wider healthcare** system including **other clinics and hospitals** view using mobile health apps for delivering cardiac care services as a **valuable strategy**?

13. When we think about next 3-5 years period, how you think the **policies and regulations** around digital technology including mobile apps for healthcare will change?

14. Do you expect to see any major changes in the near future;
    - **target population**?
    - **App use** ?
    - individual user's **opinion and perception** about the app?
15. Do you have any suggestions to improve **the adoption and continued use** of the app?
    - *Patient level*
    - *Specialist level*
    - *Institute level*

PART B: Semi structured guide for interviews with nurses

1. Tell me a bit about your role in this clinic and in relation to the mobile app.
   Prompts:
    - *For how long have you been doing this?*
    - *On average, how many people do you train a week/ month?*
    - *Do they all agree to sue the app? If not, what are some common reasons to refuse it?*
    - Can people try it out, maybe for a shorter time, before committing to it?

2. What are some common facilitators and barriers for training?
   Prompts:
    - *Patient related factors*
    - *Workplace related factors*

3. Do you think the app is designed in an appropriate manner to accommodate different socio-demographic characteristics of the target population?
   Prompts:
    - *Age*
    - *Health literacy/ digital literacy*

4. What do patients think about the app? Have they talked to you after using the app for some time?
   Prompts:
   - *Do they understand what it is for?*
   - *What are some frequent questions they ask about the app?*
   - *Have there been any complaints?*

5. In your opinion, what's the value of this app to patients?

6. What's the value of this app to you?
   Prompts:
   - *app generated data use.*
   - *information received through the app.*

7. What value does it add to the health system?

8. How did the app change the workflow and patient care for you?
   Prompts:
   - *Any additional steps to your routine workload? Was it difficult?*
   - *How do you manage additional work required to train the patients?*
   - *Were you supported with training about the app?*
   - *Do you get continuous support if you have any questions?*

9. Are there any barriers/ facilitators and broader influences for the implementation of the app?
   Prompts:
   - *Patient level factors*
   - *Work environment factors to recommend the app – what additional steps were introduced to your work in care provision because of this app implementation?*
   - *Work environment factors to use data from the app.*

10. Do you think the app captures and transmits data correctly from patients' end?

11. What do you think about the organisation's capacity and readiness to implement this app in the long run?
    Prompts:
    - *Capacity*
    - *Readiness*
    - *Any challenges for the organisation to invest in this technology in the future?*

12. Do you have any suggestions on what needs to be changed to improve its long-term use among people who require cardiac rehabilitation?
    - *training*
    - *support during the use at home*
    - *app features*
    - *anything else?*

PART C: Interview guide for individuals who used the mobile app at least once

Opening questions:

1. *If you don't mind me asking, how old are you?*
2. *How long ago were you diagnosed with atrial fibrillation?*
3. *When was your ablation surgery done?*
4. *Do you live closer to your usual clinic or very far?*
5. *How did you first find out about this mobile app?*
6. *Did you decide to give the app a try, or did you choose not to use it?*

7. Why did you decide to try the app?
   Prompts:
   - was there something specific you liked about the app?
   - do you generally enjoy trying new things?
   - do you like using apps to make life easier?

8. Did you receive instructions to download and use the app via email? How helpful was this process? Would you have preferred a face-to-face training with the nurses instead?

9. How long have you been using the app?

10. How frequently have you engaged with the app? Let's say generally in a week, how many days would you exercise as recommended by the app?

11. Did you need help using the app at home?
    - If yes, who helped you?

12. Did you use the 12-week heart rehab program in the app?
    - If yes, was it useful? Did you use it regularly?
    - If no, why not?

13. In addition to the rehab schedule, what other features of the app did you use the most?
    Prompts:
    - Exercises?
    - Reading about heart health?
    - Getting reminders?
    - Keeping track of things like your blood pressure and heart rate?

14. Have you noticed any difference in the frequency you'd get an AF related symptoms before the procedure and after?

15. Do you think the app gave you enough information to help with your decisions?
    - Was the information relevant to you?
    - Were the instructions practical?
    - Did you like how the information was presented (words, images, videos)?
    - Was the information valuable?

16. What made using the app easier for you?
    - Was it easy to navigate and find what you needed?
    - Could you personalise features (goals, reminders)?
    - Did you feel your data was private and secure?

17. How do you think your clinical team at St Andrew's use your data you insert into the app?

18. Do you think the clinical team cared about you using the app? Did they encourage you to use it?

19. Did you have any concerns about using the app?
    - Data privacy?

20. If you had to pay for this app, would you still consider using it?

21. How satisfied are you with the app?

22. Do you have any suggestions for improving the app for long-term use?
    - More training?
    - Better support for using it at home?
    - Different app features?

PART D: Interview guide for individuals who declined or did not use the mobile app

Opening questions:

1. *If you don't mind me asking, how old are you?*

2. *How long ago were you diagnosed with atrial fibrillation?*

3. *When was your ablation surgery done?*

4. *Do you live closer to your usual clinic or very far?*

5. *How did you first find out about this mobile app?*

6. *Did you decide to give the app a try, or did you choose not to use it?*

7. Can you tell me one or two main reasons why you did not want to try it?
    - Not useful for you?
    - Too busy ?
    - Too much equipment/facilities required (a mobile phone or a laptop/ tablet, internet connection, BP measuring devices that can feed the app etc) an issue?
    - Naturally not a tech person/ do not like to use technology?
    - Never used apps before

8. Did the nurses / they explain the benefits of using the app to you?

9. Do you think it would have made a difference in your decision to use the app if they had explained the benefits to you?

10. Did your doctor mention anything about the app to you?

11. Do you think it would have made a difference in your decision to use the app if your specialist doctor had explained it to you during consultations?

12. Were you satisfied with how the instructions for the app were sent to you? Do you think face-to-face training on the app might have encouraged you to try it?

13. Have you ever used any mobile health apps before?

14. If we imagine for a moment that you were using the app, what features would you like it to have? Especially features that could help with your heart condition.

15. Did anyone discuss cardiac rehabilitation with you either before or after your procedure?

16. Do you think the information you enter into the app, if you were using one, could be helpful in planning your management plan?

17. Can you think of anything would have interested you enough to give it a try? This could be related to the process of how this app was introduced to you or any factors or features about this app?
18. Is there anything else we haven’t talked about yet that you’d like to share with me about the app?
